# Viscosupplementation improves pain, function and muscle strength, but not proprioception, in patients with knee osteoarthritis: a prospective randomized trial

**DOI:** 10.6061/clinics/2019/e1207

**Published:** 2019-11-11

**Authors:** Phelippe Augusto Valente Maia, Victor Rodrigues Amaral Cossich, José Inacio Salles-Neto, Diego Pinheiro Aguiar, Eduardo Branco de Sousa

**Affiliations:** Instituto Nacional de Traumatologia e Ortopedia Jamil Haddad, Rio de Janeiro, RJ, BR

**Keywords:** Dexamethasone, Hyaluronic Acid, Osteoarthritis, Viscosupplementation

## Abstract

**OBJECTIVES:**

This study aimed to evaluate the clinical outcomes of intra-articular infiltration with hyaluronic acid and dexamethasone alone and in combination in the treatment of knee osteoarthritis (OA).

**METHOD:**

This prospective randomized trial evaluated 44 patients undergoing treatment for OA. Patients were selected through clinical and radiological analysis using the American College of Rheumatology criteria. We included patients aged between 50 and 70 years who presented with K-L stage ≤2 knee OA and normal limb alignment. Patients with a previous history of knee injury (ligamentous, meniscal or traumatic), infection, patellofemoral OA or chondroprotective drug use in the previous year were excluded. Participants were randomized into 3 groups and underwent treatment with viscosupplementation (VS, n=16), viscosupplementation plus dexamethasone (VD, n=16) or dexamethasone (DX, n=12). All patients were evaluated before and 6 weeks, 3 months and 6 months after infiltration. Analysis included a physical examination, the Western Ontario and McMaster Universities Arthritis Index (WOMAC) questionnaire (total score and domain subscores) and an evaluation of knee extensor and flexor strength and proprioception using an isokinetic dynamometer.

**RESULTS:**

VS significantly improved the WOMAC total score and subscores for pain, stiffness and function for up to 6 months after infiltration. It also improved knee extensor and flexor strength during the same period. Proprioception was not affected by any of the treatments.

**CONCLUSIONS:**

VS alone improved pain, stiffness and function according to the WOMAC total score and subscores and improved knee extensor and flexor strength, but not proprioception, for up to six months after infiltration. These findings suggest that VS has a positive effect on quadriceps arthrogenic inhibition.

## INTRODUCTION

Osteoarthritis (OA) is a degenerative disease that causes progressive joint pain and disability. Recently, OA was defined as a disease that involves the synovial joint as a whole (1). The prevalence of OA is high worldwide, estimated as 3.8% in 2010 (2). Quadriceps strength is reduced 50% in patients with knee OA (3) and represents a feature of the disease (4). This occurs partly due to a neural activation deficit of the quadriceps known as arthrogenic muscle inhibition (5,6). The conservative treatment of OA involves a set of nonpharmacological measures, including education, diet, rehabilitation, and pharmacological approaches, ranging from analgesics and anti-inflammatory drugs to infiltration agents, to promote pain relief, maintain range of motion (ROM), restore quality of life and slow the natural disease progression (7).

Corticosteroids, in their crystalline form, have been administered since the 1950s, yielding great analgesic improvements (8). Their mechanism of action involves altering B and T cell immune function and inhibiting phospholipase A2 to decrease the expression of inflammatory cytokines (8). Dexamethasone (DX) is a corticosteroid widely used in clinical practice; at a low dosage, DX exhibits chondroinductive properties *in vitro* that could be useful in modifying the course of OA (10).

Hyaluronic acid (HA) has a structure that allows joint lubrication and restores the rheological properties of synovial fluid (11). HA rose as an option for the conservative treatment of OA approximately 20 years ago due to its better clinical results compared to those of corticosteroids, mainly at 4 weeks after infiltration (12-14). Recently, it has been suggested that the addition of the corticosteroid triamcinolone hexacetonide improves the first-week symptom and function scores of viscosupplementation (VS) (13).

Hence, we hypothesized that HA combined or not with corticosteroids could help minimize the effects of quadriceps arthrogenic muscle inhibition in OA patients, as evaluated by the WOMAC total score and subscores and knee flexor and extensor proprioception and strength.

The goal of this study was to clinically evaluate the use of VS with sodium hyaluronate alone and in combination with DX for arthrogenic quadriceps inhibition in patients with knee OA.

## METHODS

This prospective randomized trial was approved by the Institutional Research Board (CAAE: 24140813.2.0000.5273), and all patients signed an informed consent form to participate.

### Patients

**Figure 1:**
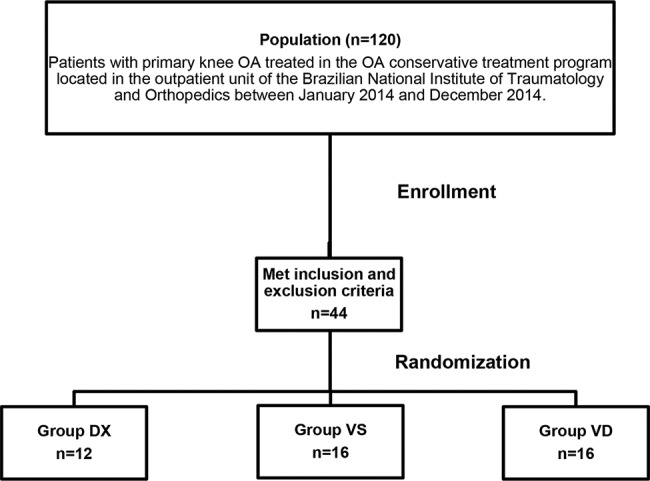
Study flow diagram.

The sample consisted of 44 patients with primary knee OA, according to the American College of Rheumatology criteria (15), who were treated in the OA conservative treatment program located in the outpatient unit of the Brazilian National Institute of Traumatology and Orthopedics between January 2014 and December 2014. This outpatient unit receives three new patients per week, and the study population consisted of these patients (120 patients). Patients over 50 years of age who presented with knee pain and radiographic knee OA classified by Kellgren and Lawrence as stage ≤2 and normal limb alignment (3° to 8° valgus) were included in the study. Patients with a previous history of traumatic injury, infection or surgery in the same knee were excluded. Patients presenting positive Lachman, anterior drawer and/or pivot-shift tests, suggesting knee instability, and positive meniscal tests, suggesting meniscal tear, were also excluded. Finally, we excluded patients with a body mass index (BMI) over 35 kg/m^2^, patients with patellofemoral arthritis, identified on radiographs, and patients who had a history of treatment with chondroprotective drugs, corticosteroid infiltration or VS in the previous year (Figure 1).

### Clinical procedures

Patients were prospectively selected in the outpatient unit and designated for clinical evaluation in the clinical research unit of the institute. In this evaluation, patients received information about the research, including the risks and benefits. The patients who met the eligibility criteria and agreed to participate signed the informed consent form to be included in the study. After randomization and group allocation, patients were interviewed and submitted to clinical evaluation and infiltration. Patients were evaluated immediately before and 6 weeks, 3 months and 6 months after infiltration. The protocol was not registered with any clinical trial platform.

Randomization was performed using blocks of six patients (two for each group) and sealed envelopes containing the acronym identifying the groups (VS/VD/DX), which was opened after completing the clinical evaluation. Infiltration was performed by a member of the staff (PAVM) who did not participate in the evaluations. After allocation, the patients were treated as follows:

1. Viscosupplementation group (VS group): VS alone with 3 doses (6 mL) of sodium hyaluronate 15 mg/mL (Orthovisc^®^, Johnson & Johnson^®^) in a single shot.
2. Viscosupplementation plus dexamethasone group (VD group): VS with 3 doses (6 mL) of sodium hyaluronate 15 mg/mL (Orthovisc^®^, Johnson & Johnson^®^) plus 1 dose of DX (4 mg/1 mL) in a single shot.
3. Dexamethasone group (DX group): 1 dose of DX (4 mg/1 mL) in a single shot.

Evaluations were performed by other members of the staff who did not participate in the infiltration procedures (EBS and VRAC). Infiltration was performed with the patient seated and knees flexed through anterolateral access to the knee. To evaluate arthrogenic quadriceps inhibition, the protocol included clinical, proprioception and knee extensor and flexor strength evaluations.

### Clinical evaluation

Age (in years), laterality (right/left, self-reported by preferred limb to kick a ball regardless of the influence of OA), weight (in kilograms), height (in meters), BMI (kg/m^2^), knee joint ROM and lower limb alignment were recorded in the clinical evaluation.

Radiographic analysis included a standing full-length lower extremity, an anteroposterior (AP) and lateral view of the knee joint to stratify the OA severity according to the Kellgren-Lawrence classification and measurement of the lower limb mechanical axis.

The subjective functional evaluation used the Western Ontario McMaster Universities Osteoarthritis Index (WOMAC) questionnaire validated for the Portuguese language (16), including the total score and its subscores for the pain, stiffness and function domains.

### Proprioception evaluation

The knee proprioception evaluation involved sensing of the joint position. An isokinetic dynamometer (CSMI, Humac Norm) was used in all of these procedures. All tests were conducted with the subject in a seated position with the hip at approximately 90° and the entire back touching the backrest. The patient was fixed to the seat by means of a belt.

First, the subject was required to experience and then reproduce joint positions, through voluntary movements in both cases. Two target positions were used: 20% and 50% of the flexion-extension (FLEX-EXT) ROM (0%=maximum extension). Throughout the procedure, the patient was blindfolded. Variations of ±5° around the target position were allowed. If this margin was violated, the trial was discarded, and a new attempt was made. A total of 10 trials were performed, and 5 were chosen randomly for each target position. The individual error value for each attempt was determined through the difference between the position reproduced and the position experienced. The proprioceptive performance was determined with the absolute error (AE), obtained through the arithmetic mean of the absolute values of individual errors. The commands for carrying out the task were issued verbally by the evaluator, and the direction of movement was always from flexion to extension. During the procedure, the subject was blinded with special goggles to attenuate visual bias.

### Knee extensor and flexor strength evaluation

After 10 minutes of rest, the strength evaluation was carried out. Prior to each test, all calibration procedures were accomplished, and the participant performed a warm-up familiarization process comprising movements of knee extension and flexion at submaximal effort. Each test consisted of one set of five repetitions in the isokinetic concentric-concentric mode at an angular speed of 60°/s, and only the involved limb was evaluated. The maximum value found was defined as the peak torque (PT), which was normalized by the subject’s weight and recorded for statistical proposes.

### Statistical analysis

The results are presented as the mean and standard deviation (SD). For the WOMAC scores, comparisons were made within groups by one-way ANOVA at each measurement time point (PRE, 6 W, 3 M and 6 M). The dependent variables were PT EXT, PT FLEX, AE20 (absolute error at 20% of FLEX-EXT ROM), AE50 (absolute error at 50% of FLEX-EXT ROM), and the WOMAC total score and pain, stiffness and physical activity subscores. The Shapiro-Wilk test showed all variables to be normally distributed (all, *p*>0.05). To compare demographic data (height, weight, BMI, and age), one-way ANOVA was used. First, one-way repeated measures ANOVA was used to compare within-subject effects over time (PRE, 6 W, 3 M, and 6 M) and between-treatment effects (DX, VD and VS). When a significant within-main effect was observed, one-way ANOVA was performed again for each group separately. If sphericity could not be assumed, the Greenhouse-Geisser correction was applied. Pairwise post hoc comparisons were carried with Bonferroni’s test. To preserve the repeated measures design if any evaluation session throughout the study was lost, the respective subjects data were withdrawn from the analysis. The effect size was assessed by *η_p_*^2^ and classified as small=0.01, medium=0.06, or large=0.14 (17). All calculations were conducted using IBM SPSS software for Windows (EUA) and graphics produced with GraphPad Prism (EUA). The significance level was set at 0.05.

## RESULTS

### Clinical data

Initially, forty-four patients were included in the study, and the demographic data are presented in Table 1. There were no significant differences regarding the demographic data, except for height, which was greater in the DX group than in the VD group.

**Figure 2:**
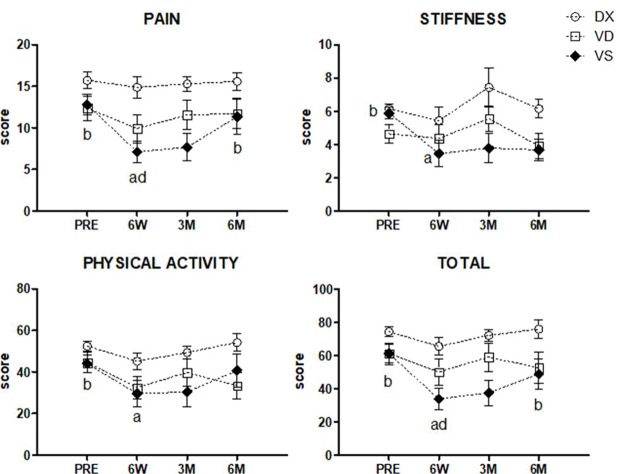
**WOMAC score evaluation.** Significant effects were observed over time in the WOMAC total score and pain, stiffness and physical activity subscores (all, *p*<0.05). Additionally, significant effects between groups were observed in the pain (*p*=0.03), stiffness (*p*=0.03), and physical activity (*p*=0.03) subscores, but not in the WOMAC total score (*p*=0.07). Pairwise verification demonstrated differences between DX and VS. Further separate analysis revealed significant differences only within the VS group in terms of the pain (*p*=0.01), stiffness (*p*=0.04), and physical activity (*p*=0.05) subscores and the WOMAC total score (*p*=0.03). Symbols represent mean values, dashed and straight lines represent standard deviations. 6 W: six weeks, 3 M: three months and 6 M: six months; a: compared to PRE; b: compared to 6 W; c: compared to 3 M; d: compared to 6 M.

Statistically significant effects were observed over time regarding the WOMAC total score and pain, stiffness and physical activity subscores (all, *p*<0.05). Additionally, significant effects between groups were observed in the pain (*p*=0.03, *η_p_*^2^=0.24), stiffness (*p*=0.03, *η_p_*^2^=0.28), physical activity (*p*=0.03, *η_p_*^2^=0.26) subscores, but not in the total score (*p*=0.07, *η_p_*^2^=0.18). Pairwise verification demonstrated a difference between the DX and VS groups. Further separate analysis revealed significant differences only within the VS group regarding the pain (*p*=0.01, *η_p_*^2^=0.39), stiffness (*p*=0.04, *η_p_*^2^=0.29), and physical activity (*p*=0.05, *η_p_*^2^=0.33) subscores and the total score (*p*=0.03, *η_p_*^2^=0.37) (Figure 2 and Table 2).

### Proprioception evaluation

**Figure 3:**
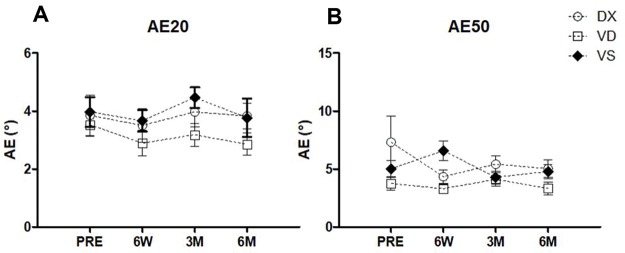
**Proprioception evaluation.A. Absolute error 20%.** There were no significant differences regarding the AE20 over time (*p*=0.30) or between groups (*p*=0.20). **B. Absolute error 50%.** The same results were found for the AE50 over time (*p*=0.35). General analysis showed a significant difference between groups (*p*=0.036), which was not confirmed by pairwise comparison.

There were no significant differences in either the AE20 (within *p*=0.30, *η_p_*^2^=0.04; between *p*=0.20, *η_p_*^2^=0.20) or the AE50 (within *p*=0.35, *η_p_*^2^=0.04; between *p*=0.036, *η_p_*^2^=0.21) (Figure 3A). General analysis of the AE50 revealed a significant effect between groups. However, pairwise comparisons did not identify significant differences (Figure 3B).

### Strength evaluation

**Figure 4:**
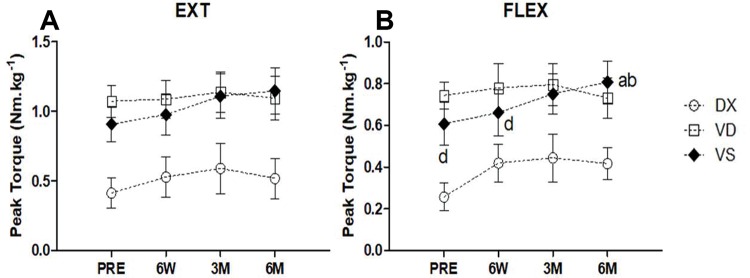
**Strength evaluation. A. Knee extensor peak torque.** No significant differences regarding PT EXT over time (*p*=0.07) were identified. Analysis between groups presented significant differences (*p*=0.02). Pairwise differences were observed between DX and VD (*p*=0.027) and between DX and VS (*p*=0.043). **B. Knee flexor peak torque.** Regarding PT FLEX, significant main effects were observed both over time (*p*=0.01) and between groups (*p*=0.02). A pairwise significant difference was observed for DX and VD (*p*=0.034), but not for DX and VS (*p*=0.067). Further separate analysis revealed significant differences in both PT EXT (*p*=0.03) and PT FLEX (*p*=0.03) only in the VS group. Symbols represent mean values, dashed and straight lines represent standard deviations. 6 W: six weeks, 3 M: three months and 6 M: six months; a: compared to PRE; b: compared to 6 W; c: compared to 3 M; d: compared to 6 M.

We found no significant differences in PT EXT (Figure 4A) in any of the groups over time (*p*=0.07, *η_p_*^2^=0.10). However, when the groups were compared, differences were identified (*p*=0.02, *η_p_*^2^=0.28). Moreover, pairwise differences were observed between DX and VD (*p*=0.03) and between DX and VS (*p*=0.04). Regarding PT FLEX (Figure 4B), a significant main effect was observed over time (*p*=0.01, *η_p_*^2^=0.16) and between groups (*p*=0.02, *η_p_*^2^=0.26). A pairwise significant difference was observed between DX and VD (*p*=0.03), but not between DX and VS (*p*=0.07). Further separate analysis revealed a significant difference in both PT EXT (*p*=0.03, *η_p_*^2^=0.31) and PT FLEX (*p*=0.03, *η* *p*2=0.54) only for VS.

### Adverse effects

None of the patients included in the study presented adverse effects.

## DISCUSSION

Arthrogenic quadriceps inhibition is present in patients with knee OA and is associated with joint inflammation, pain, and swelling, contributing to muscle atrophy and hindering rehabilitation (6). We hypothesized that HA alone or in combination with corticosteroids could help minimize quadriceps muscle arthrogenic inhibition and help improve the nonoperative treatment of knee OA.

We designed a prospective randomized trial to compare the use of DX and HA alone and in combination in the treatment of knee OA by evaluating the WOMAC total score and subscores and knee extensor and flexor strength and proprioception. Our results show that VS alone significantly improved pain, stiffness and function, as shown by the WOMAC subscores. In addition, VS alone improved knee extensor and flexor strength, but not proprioception.

Intra-articular corticosteroid use is criticized because of its short analgesic effect, which lasts for up to approximately one month after infiltration (18), in addition to not having any modifying effect on the disease. Nevertheless, its use is recommended in many protocols for OA treatment (19). HA reestablishes the ideal rheological properties of synovial fluid but may cause synovitis after infiltration and does not produce an immediate analgesic effect; however, it is more effective in the long term, with effects lasting up to six months (18). Nevertheless, a study confirmed its efficacy by reporting a reduction in pain of more than 50% in most patients treated with infiltration (20). The association between corticosteroids and HA has been recently explored to enhance the clinical results of each drug class (13).

One study showed significant improvement in knee pain and function in patients treated with HA infiltration compared to placebo at five weeks after treatment. However, these effects lasted only until the 25^th^ week after infiltration (14,21-22). Another study used three different kinds of HA in women between 40 and 60 years old with moderate knee OA or degenerative meniscal tear, and similar to our study, the study concluded that HA is in fact the best conservative treatment for these patients (23). Major efficacy of HA was confirmed at 8 weeks after infiltration in a systematic review including 7 studies with 606 patients (24). Another randomized and double-blind study with 51 patients verified that the clinical effects of HA and corticosteroids as a local therapy are comparable and that both are useful for the conservative treatment of OA (25). In our study, three doses of isolated HA in a single shot improved knee pain, stiffness and function as evaluated by the WOMAC total score and subscores. In addition, knee extensor and flexor strength was increased for up to six months in patients who received HA infiltration alone. We believe this was due to pain reduction, which allowed patients to perform rehabilitation appropriately. However, we found no differences in proprioception parameters.

The use of a single shot of HA or methylprednisolone in patients with mild OA was compared. Similar clinical results were observed between the groups, but HA showed superior analgesic effects after the 12^th^ week (26). However, in a multicentric, double-blind, randomized study evaluating 391 patients, HA presented no analgesic superiority over corticosteroids (22). The effects of VS with Hylan G-F 20 and triamcinolone alone and in combination were also compared. In one study, this combination improved pain, mainly in the first week after infiltration (9), while in another study, the improvements in the functional scores lasted up to three months (27). Another study involving 47 patients showed that the same combination improves subjective pain and function (28). In the same way, another clinical study demonstrated similar analgesic improvement through a combination of corticosteroids and HA compared to HA alone. In addition, magnetic resonance imaging did not detect radiographic progression of the disease during the study period in either group (29). In this study, three doses of isolated HA in a single shot improved knee pain, stiffness and function, as indicated by the WOMAC scores. In addition, knee extensor and flexor strength was increased for up to six months in patients who received infiltration with HA alone. We believe this was due to pain reduction, which allowed patients to perform rehabilitation appropriately. However, we found no differences in proprioception parameters.

Initially, it was demonstrated that proprioception was affected by knee OA and that HA infiltration did not negatively affect proprioception (30). Then, in a prospective, randomized, placebo-controlled trial involving 63 patients with grade II-III knee OA, an increase in isokinetic strength and proprioception after infiltration with Hylan G-F 20 was demonstrated (31). Finally, another study including patients between 50 and 70 years old with bilateral knee OA verified an increase in postural stability and a decrease in the risk of fall in patients treated with HA infiltration compared to worsening conditions in those who received the placebo (32). It has been previously demonstrated that aspirating joint effusion and/or injecting a local anesthetic into the joint abolishes arthrogenic muscle inhibition (33-34). Therefore, we hypothesized that injecting corticosteroids alone or in combination with HA could have the same clinical effect. Our data indicate improvement in knee extensor and flexor strength lasting for up to six months in patients treated with HA alone and in knee extensor strength in patients treated with HA combined with DX. The magnitude of this effect was greater in the HA group, which confirmed our hypothesis that HA could modulate arthrogenic inhibition in patients with knee OA.

The importance of strength training has been widely proven, with the consequent development of strength (35-36) and proprioception in patients with OA (36). Healthy knees and knees in early stages of OA present greater quadriceps and hamstring strength than those in late stages of the disease (37). Hence, quadriceps strength training could reduce pain and increase proprioception, reinforcing the importance of muscle strength training in patients with knee OA (38). Similarly, a metanalysis concluded that proprioception exercises are effective in the conservative treatment of knee OA (39).

The main strength of this study is that we compared knee OA treatment using VS alone, VS combined with corticosteroids and corticosteroids alone using the same protocol. Moreover, the evaluation was performed using not only scores but also the findings of knee flexor and extensor strength and knee proprioception evaluations performed using an isokinetic dynamometer.

The limitations of the study include the small sample number and the availability of rehabilitation for the patients included in the study. All patients received guidance regarding the rehabilitation protocol to which they should have access, considering the protocol used in our institution and those available in our public health system. There is a lack of scientific evidence in the literature regarding the effect of HA on patellofemoral OA. Thus, these patients were excluded to avoid misinterpretation of the data.

In conclusion, according to our results, VS alone improved pain, stiffness and function according to the WOMAC total score and subscores for up to six months after infiltration. VS also improved knee extensor and flexor strength in the same period, but not proprioception. These findings suggest that VS has a positive effect on quadriceps arthrogenic inhibition.

## AUTHOR CONTRIBUTIONS

Maia PAV was responsible for the concept and design, provision of study material or patients, collection and assembly of data, data analysis and interpretation, manuscript writing, and approval of the final version of the manuscript. Cossich VRA was responsible for the collection and assembly of data, data analysis and interpretation, and approval of the final version of the manuscript. Salles-Neto JI was responsible for the data analysis and interpretation, and approval of the final version of the manuscript. Aguiar DP and Sousa EB were responsible for the concept and design, collection and assembly of data, data analysis and interpretation, manuscript writing, and approval of the final version of the manuscript.

## Figures and Tables

**Table 1:** **Demographic characteristics of patients included in the study.** Demographic data of patients in each group.

	Demographic data		
	DX	VS	VD
Patients (n, F:M)	12 (11:1)	16 (10:6)	16 (10:6)
Laterality (R/L)	9/3	10/6	7/9
Height (m)	156.7±1.98	1.63±3.2	165.6±2.2*
Weight (kg)	76.8±3.9	86.5±4.9	79.3±3.2
BMI (kg/m^2^)	31.4±1.8	31.9±1.3	29.0±1.2
Age (years)	60.3±1.7	56.6±1.0	54.5±2.4

DX: dexamethasone; VS: viscosupplementation; VD: viscosupplementation plus dexamethasone, BMI: body mass index, F: female; M: male; R: right; L: left.

* *p*<0.05 *vs*. DX group.

**Table 2:** **WOMAC questionnaire data.** Data from the WOMAC questionnaire were divided into the score for three domains (pain, stiffness and function) and the total score for all three groups.

Group	PRE	6 W	3 M	6 M	F-value	*p*-value	*η_p_*^2^
**WOMAC - Pain**							
VS	15.3 (2.8)	14.4 (3.5)	14.3 (3.6)	15.4 (2.6)	5.09	**0.01**	0.39
VD	12.4 (4.9)	9.9 (5.7)	11.6 (5.9)	11.7 (6.0)	0.83	0.46	0.08
DX	12.8 (3.8)	7.1 (3.8)	7.1 (3.9)	11.3 (6.2)	0.14	0.93	0.02
**WOMAC - Stiffness**							
VS	7.3 (3.2)	5.5 (2.0)	7.0 (3.1)	6.3 (1.4)	3.22	**0.04**	0.29
VD	4.6 (1.9)	4.4 (2.7)	5.6 (2.6)	3.9 (2.6)	0.15	0.15	0.18
DX	5.9 (1.1)	3.4 (2.4)	3.8 (2.6)	3.7 (1.9)	1.17	0.34	0.16
**WOMAC - Physical Activity**							
VS	52.4 (5.8)	45.1 (11.0)	49.3 (7.7)	54.1 (11.4)	3.90	**0.02**	0.34
VD	44.1 (14.8)	35.7 (19.1)	41.9 (20.5)	37.0 (22.7)	2.26	0.15	0.18
DX	42.4 (14.1)	23.2 (13.3)	26.1 (16.3)	33.8 (21.5)	1.17	0.34	0.16
**WOMAC - Total**							
VS	68.4 (18.3)	59.4 (21.3)	67.4 (15.9)	73.5 (15.4)	4.71	**0.03**	0.37
VD	61.1 (21.1)	50.0 (27.2)	59.0 (28.4)	52.6 (31.1)	1.04	0.34	0.09
DX	61.1 (16.9)	33.8 (19.1)	37.6 (23.1)	48.8 (28.0)	1.08	0.37	0.15

DX: dexamethasone; VS: viscosupplementation; VD: viscosupplementation + dexamethasone; PRE: before treatment; 6 W: six weeks after treatment; 3 M: three months after treatment; 6 M: 6 months after treatment. Values are shown as the mean (SD). Significant differences are in bold.
